# Applying a quantitative fire risk assessment method in hospital settings: Case study

**DOI:** 10.1371/journal.pone.0315936

**Published:** 2025-02-06

**Authors:** Marzieh Beljikangarlou, Ali Ghazvinloo, Alireza Dehdashti, Mohammad Reza MirLavasani

**Affiliations:** 1 Student Research Committee, Semnan University of Medical Sciences, Semnan, Iran; 2 Faculty of Medical Sciences, Department of Occupational Health and Safety, Tarbiat Modarres University, Tehran, Iran; 3 Department of Environmental Management, Science and Research Branch, Islamic Azad University, Semnan, Iran; 4 Social Determinants of Health Research Center, Semnan University of Medical Sciences, Semnan, Iran; 5 Department of Occupational Health and Safety, School of Health, Semnan University of Medical Sciences, Semnan, Iran; 6 Faculty of Natural Resources and Environment, Department of Environmental Management, Science and Research Branch, Islamic Azad University, Semnan, Iran; SKUMS: Shahrekord University of Medical Science, ISLAMIC REPUBLIC OF IRAN

## Abstract

Understanding and mitigating fire risks in healthcare settings are crucial for ensuring the safety of individuals, especially during the current pandemic, which has increased the use of oxygen-supply equipment and potentially raised fire hazards. This case study, conducted in two university hospitals in Semnan, Iran, examined fire risk factors in healthcare facilities using a developed fire risk assessment method. A total of 28 wards and 74 compartments were assessed. Data collection included topographical structure analysis, building usage evaluation, and process documentation review. The FRAME method, validated in previous studies, was used to calculate fire risk levels for buildings, contents, occupants, and activities. The fire risk assessment revealed varying risk levels across different wards and compartments in the two hospitals. Hospital A exhibited higher fire risk levels compared to Hospital B, with several wards in Hospital A classified as "High" or "Very High" risk. Factors contributing to higher risk levels included building design, occupancy density, and the presence of flammable materials. Occupants in certain wards, particularly those with high occupancy rates and limited evacuation routes, were identified as being at increased risk. Activities such as the storage and handling of flammable materials were also found to contribute to elevated risk levels in specific areas. The study emphasizes the importance of implementing targeted fire safety measures, especially regarding oxygen-supplying equipment, high-density ventilators, and limited escape routes, to mitigate risks effectively in healthcare settings. This comprehensive assessment can guide best practices in fire safety management in hospitals.

## 1. Introduction

### 1.1 Background and significance

Hospital settings should be able to provide health-care services to affected people in disasters and emergencies without interruption [1]. Across countries, fire and explosion hazards in the hospitals and healthcare facilities have caused damage to people, properties, work processes, and the environment [2,3]. In Iranian hospital environment, prior investigations have shown that medical equipment, oxidizing agents, and operations are associated with high risk of fire and explosion [4–6].

A tragic incident in an Iraqi hospital in April 2021, where an oxygen cylinder explosion caused the deaths of 82 people, shows the severity of the problem [7]. Many equipment and operations in hospital environments are associated with a higher risk of fire and explosion. Recent research reported that hospitals and healthcare facilities worldwide must take necessary precautions to prevent incidents, as the potential for fire-related disasters exists due to both equipment and environmental factors [8,9]. The literature review of fire risk assessment, fire, and explosion in hospitals and healthcare settings in Iran emphasizes the critical need for risk assessment strategies, effective emergency evacuation plans, and enhanced safety measures within healthcare facilities [10,11]. Overall, all the hospitals and health-care facilities have legal responsibility to create a safe environment for both workers and patients. Observing safety considerations in different wards of hospitals can lead to reduction of risks and probable accidents [6]. The first step for prevention and mitigation the risks and harmful consequences is risk assessment as earlier studies pointed to the rational of risk assessment approaches for determining levels of hazard and prioritizing them for removing [12,13].

### 1.2 Fire risk variations Across hospital wards

In Iranian hospitals, fire and explosion hazards are particularly concerning due to factors like flammable materials, faulty electrical installations, and inadequate fire safety measures [10]. Assessments of Iranian hospital safety scores reflect these challenges, with a study of 224 hospitals showing an average safety rating of just 32.4 out of 100, and over half of the hospitals were categorized as "low safe" [14]. There is an urgent need for better mitigation strategies, including the establishment of national safety committees and the implementation of comprehensive fire safety management protocols. Without these measures, the fire risk in Iranian hospitals remains alarmingly high, threatening the safety of both patients and healthcare staff [14,15]. Different hospital wards are associated with varying levels of fire risk, depending on the specific activities and materials handled in these areas. Intensive care units (ICUs), operating rooms, and laboratories are considered high-risk wards due to the extensive use of flammable materials, oxygen therapy, and sensitive medical equipment [16,17]. In ICUs, for example, the increased use of oxygen therapy significantly elevates the fire risk, as oxygen can accelerate combustion [8,9]. Similarly, operating rooms are vulnerable because of the presence of flammable anesthetic gases, surgical equipment, and electrical systems [18]. Laboratories face risks due to the handling of hazardous chemicals and [19,20]. Past studies have demonstrated that these wards require tailored fire risk management strategies to address their unique hazards and mitigate potential disasters [6,21].

Although the COVID-19 pandemic highlighted fire risks related to oxygen therapy and increased use of medical equipment in ICUs, it is important to recognize that fire hazards in healthcare facilities are an ongoing concern, irrespective of pandemic conditions. Hospitals continuously rely on oxygen and other flammable materials, and medical environments remain vulnerable to fire hazards due to faulty electrical installations, inadequate fire safety protocols, and improper handling of oxygen cylinders and other medical gases [16]. Therefore, even as the pandemic has subsided, fire risk management continues to be of critical importance in ensuring the safety of hospital environments. Unsafe work practices in handling oxygen cylinders and other medical gases have been identified as contributing factors [21].

Many equipment and operations in hospital environment are associated with higher risk of fire and explosion. Scientific evidence indicates that incidents of oxygen-related hospital fires in different countries throughout the world have caused over 200 deaths during Covid-19 pandemic [16]. Intensive care units involve equipment and procedures to deliver oxygen therapy for patients who need large volumes and help keeping the air sacs in their lungs open [7,8]. Earlier studies reported increasing demand for oxygen therapy procedure in severely ill Covid-19 patients was associated with a significant increase in the risk of fire and explosion particularly due to unsafe work practices [9,21]. Considering the specific fire risks associated with different hospital wards, fire safety protocols need to be adapted and applied to each ward’s unique requirements. Comprehensive fire risk management should include tailored preventive measures for high-risk areas like ICUs, operating rooms, and laboratories, as well as general safety strategies for other parts of the hospital [12].

### 1.3 Justification for the FRAME method in fire risk assessment

Several methods have been applied to assess fire risk in hospital settings, each with its own strengths and limitations. For instance, Failure Mode and Effects Analysis (FMEA) and Multi-Criteria Decision-Making (MCDM) have been widely used to identify potential risks and assess safety measures [10]. However, these methods rely heavily on subjective judgments, and their accuracy may vary depending on the expertise of the evaluators [22,23]. The Fire Safety Engineering Approach (FSEA) provides a more technical framework but often requires extensive data inputs and can be costly in terms of time and resources [24]. Other qualitative methods, such as risk matrices, may lack the precision necessary for high-risk environments like hospitals [25–27].

In this study, the Fire Risk Assessment Method for Engineering (FRAME) was chosen as the primary tool for assessing fire and explosion risks in hospitals. Comprehensive Fire Risk Assessment Method for Engineering (FRAME) applies systematic, practical and transparent computational methods to assess the risk of fire in buildings. FRAME method considers fire exposure, fire threat, and fire protection, which can provide an adequate safety strategy against fire and explosion hazards [28]. This method does not rely on individual perception to determine the scales of indicator and the results of assessment have adequate objectivity. Accuracy, short-term execution and low execution costs are the main advantages of this method [29]. The FRAME (Fire Risk Assessment Method for Engineering) method has proven to be a highly effective and objective approach to evaluating fire risks in buildings. It offers significant advantages over traditional methods by considering fire exposure, fire threat, and fire protection measures in an integrated and transparent manner. This method is a tool to evaluate fire prevention measures, fire detection and firefighting equipment and also help fire protection engineers to define suitable and cost-effective fire protection solutions for new or existing buildings where both time and resources are limited [30,31].

### 1.4 Aims of the study

This study employed the comprehensive Fire Risk Assessment Method for Engineering (FRAME) to assess fire hazards within hospital environments. while the FRAME method has indeed been applied in other studies, our study builds upon and expands its application in several key ways. By applying this method across various healthcare facilities, the research aimed to:

1) Identify and compare fire hazards across various hospital wards, 2) Prioritize risks and implement cost-effective mitigation strategies, and 3) Determine best practices in fire risk management for preventing accidents, injuries, and potential loss of life. The study’s main goal was to contribute to the enhancement of safety protocols and the reduction of fire and explosion hazards in healthcare settings, ensuring the safety of both patients and healthcare workers.

## 2. Materials and methods

### 2.1. Study design

This study was designed as a case study, carried out in two university hospitals, from April to June 2021, during the COVID-19 pandemic. The aim was to assess fire risk using the Comprehensive Fire Risk Assessment Method for Engineering (FRAME) method. The study was conducted in two university hospitals (Hospital A and Hospital B). Hospital A with an area of 25000 m^2^ has a capacity of approximately 220 beds, distributed across 14 wards and 39 compartments including specialized units such as intensive care, emergency, surgery, pediatric, and gynecology wards. Hospital A houses a variety of essential equipment, particularly in intensive care and emergency units where oxygen-supply and ventilation systems are frequently used. Hospital B with an area of 11000 m^2^ has a capacity, with around 110 beds across 14 wards and 35 compartments, covering general care and some specialized units like obstetric and neonatal care.

In our study, the two university hospitals selected for this fire risk assessment study were chosen based on their large scale, the range of healthcare services provided, and their differing structural designs and operational complexities. Logistical possibility and access to comprehensive data also influenced the selection of these two hospitals. These factors allowed for the assessment of varied fire risk scenarios, ensuring the applicability of our findings to a broader range of healthcare environments. Also, the increased use of oxygen-supply equipment during the pandemic in these hospitals presented a significant variable in fire risk, making them model case studies for the fire risk investigation.

### 2.2. Data collection

This study used a structured approach to data collection, combining direct observations, interviews with hospital staff, and analysis of hospital records to gather information on fire risk factors across different wards. Observations were conducted systematically, guided by a checklist developed in alignment with FRAME assessment criteria, focusing on the layout, equipment storage, accessibility of emergency exits, and presence of fire protection systems in each ward. Additionally, interviews were conducted with facility managers, maintenance staff, and ward supervisors to gain insights into safety protocols, staff training, and equipment handling procedures. These semi-structured interviews were designed to capture both standardized information and contextual details unique to each department. Interviews were conducted on-site, with responses documented in a structured format for consistency.

#### 2.2.1 Topographical and structural data collection

**Fig 1:**
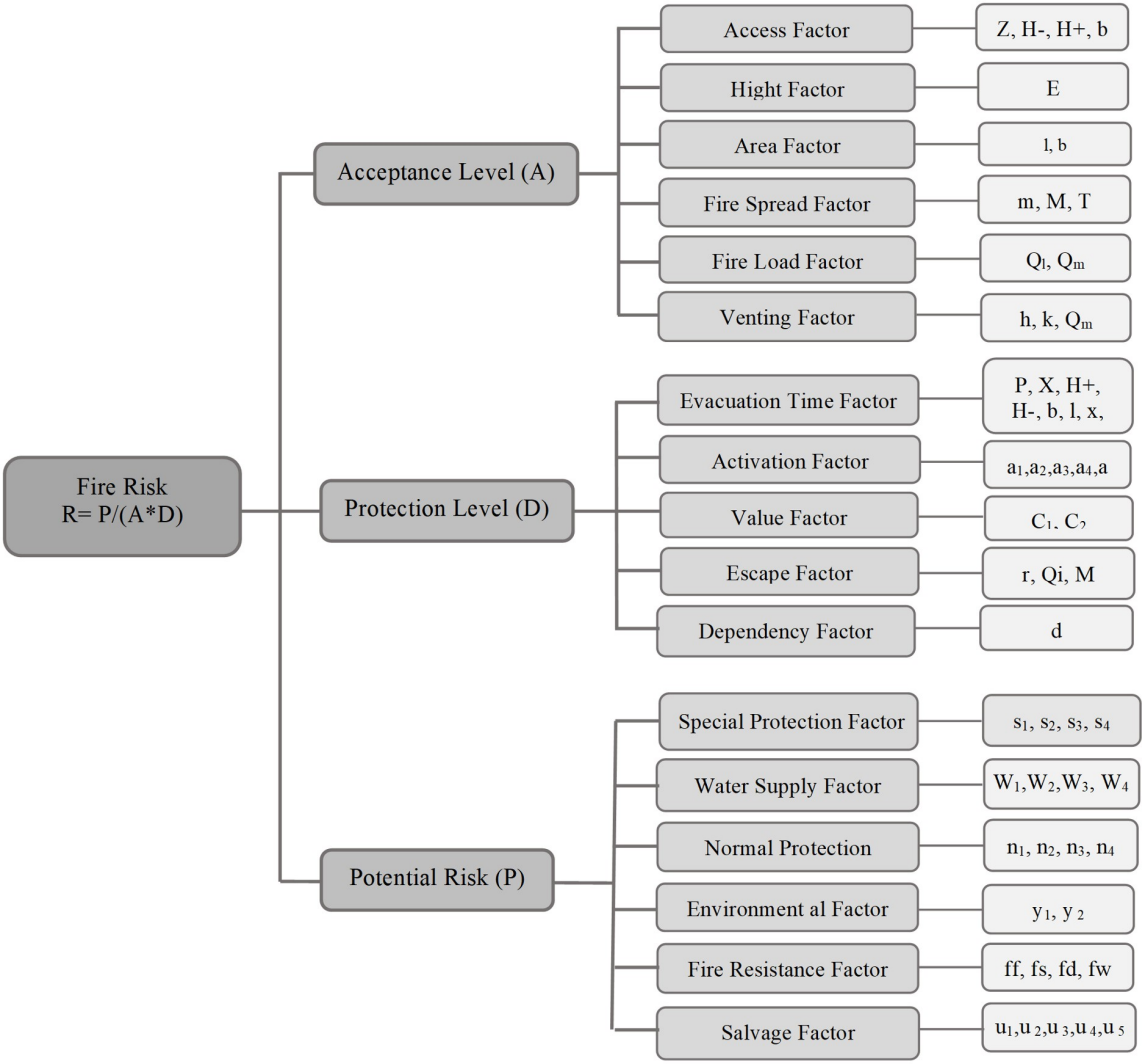
Parameters affecting the calculation of fire risk level.

Initially, detailed topographical data were collected for each hospital, including the structure of rooms, ward height from the ground, fire penetration and propagation possibilities, building usage, and space utilization. Specific focus was placed on work practices and equipment used in each ward, particularly those related to oxygen therapy during the pandemic which is known to elevate fire risks. Fig 1 illustrates the key parameters influencing the calculation of fire risk levels. These factors include structural characteristics, spatial layout, and operational practices within hospital wards, all of which contribute to the overall assessment of fire risk.

#### 2.2.2 Inclusion criteria

We established specific inclusion and exclusion criteria for selecting hospital units. Units were included if they demonstrated patient occupancy levels exceeding 50% at the time of assessment, actively used oxygen-supply equipment or high-density ventilators, or engaged in critical care activities. We included intensive care units (ICUs), emergency departments, surgical wards, pediatric wards, and other high-occupancy areas, all of which represent heightened fire risk due to the complexity of care provided. Additionally, we involved units located in buildings over five years old, aiming to capture risks associated with older facility layouts and structural features.

### 2.3. Application of FRAME method

#### 2.3.1 Risk parameter selection

The quantitative FRAME method was employed to compute fire risk scores for building structures, hospital contents, occupants, and activities. This method involves evaluating the likelihood of fire occurrence and its consequences, factoring in elements like the quantity and type of combustible materials, ignitability, and fire protection measures. Data collection was conducted using a custom-designed on-site survey checklist, developed according to FRAME guidelines. Data were gathered through direct observation, interviews with hospital personnel, technicians, and analysis of hospital process documents.

#### 2.3.2 Data analysis

We used Microsoft Excel (Version 2016) to manage and organize the data collected from topographical structure analysis, building usage evaluation, and process documentation. A computational EXCEL sheet was developed for processing data, facilitating a swift and accurate evaluation. Calculations were conducted based on the formulae outlined in Table 1, enabling a precise risk evaluation.

**Table 1:** Equations for fire risk assessment and expected building damage in hospital fires.

No.	Equation	Sub-Equation	Fire Risk Score	Percent of damage
I	R = P/A×D	P = q × i × g × e × v × z	Up to 1	10% or less
		A = 1.6 − a − t − r		
		D = W × N × S × F	1–1.3	10 to 20%
II	R_1_ = P_1_/A_1_×D_1_	P_1_ = q × i × e × v × z		
		A_1_ = 1.6 − a − t − r	1.3–1.5	20 to 30%
		D_1_ = N × U		
III	R_2_ = P_2_/A_2_×D_2_	P_2_ = q × i × e × v × z	1.5–1.7	30 to 50%
		A_2_ = 1.6 − a − c − d		
		D_2_ = W × N × S × Y	1.7–1.9	50 to 80%
IV	q = 2.3×log (Q_i_-Q_m_)-0.55			
V	v = 0.84+(0.1×log Q_m_)-√k×√h		> 1.9	80 to 100%
VI	z = 0.05×│b/z×20 +H^+^/25 or H^-^/3│			
VII	a = ∑a_i_			

#### 2.3.3 Fire risk evaluation for buildings, occupants, and activities

Separate assessments were conducted for fire risks associated with hospital buildings, contents, occupants, and activities. This was performed using equations derived from the FRAME [32] and Swiss Gretener methods [33] as detailed in Table 1. Factors such as the number of combustible materials, occupancy levels, spatial layout, and fire control systems were considered for each element. For building risks, potential damage was estimated based on fire risk scores, with hypothetical building damage percentages calculated (Table 1). For occupants, vulnerability scores were assessed based on room layout and the ease of emergency evacuation, while activity risks were evaluated by considering the types of medical procedures performed, especially those involving medical gases.

Equation I calculate the potential risk (P) based on several factors including the quantity of combustibles (q), their ignitability (i), the likelihood of fire spread (g), the presence of fire protection measures (e), the vulnerability of occupants (v), and the influence of zoning (z). This potential risk is then compared to the acceptable risk (A) level, which considers factors such as accessibility (a), time (t), and the presence of rescue teams (r), to determine the protection level (D) needed to mitigate the risk. Equations II and III are similar, focusing on the potential risk (P_1_ and P_2_) related to occupants and activities respectively, and calculating the corresponding protection levels (D_1_ and D_2_). These equations account for factors specific to occupants and activities, such as the number of occupants (N), their vulnerability (U), the space layout (S), and the type of activity (Y).

Equations IV, V, and VI introduce additional factors such as the logarithmic relationship between fire intensity (Qi) and minimum fire intensity (Qm), the effect of ventilation (v) on fire spread, and the influence of structural features (z) on fire risk. Finally, Equation VII sums up the contributions of various sub-parameters (ai) to the overall fire risk, providing a comprehensive assessment of the risk level in different areas of the hospitals. Each fire risk score is compared to an acceptable risk level (A) to determine the degree of protection needed (D). These scores are then analyzed to identify the most influential factors contributing to fire risk in each ward. Factors with scores above 1 were considered high-risk and are noted as the primary influences on fire risk in this study. Overall, these equations provide a systematic and quantitative approach to assessing fire risk, allowing for the identification of high-risk areas and the implementation of targeted mitigation measures to enhance fire safety in hospital settings. Based on the fire risk score the hypothetical destruction of the building is estimated (Table 1).

#### 2.3.4 Fire risk scoring and control measures

The FRAME method assigns a fire risk score on a four-point scale: Low, Moderate, High, and Very High. Table 2 outlines these levels and the corresponding control measures to mitigate fire risk. For example, a "Low" risk score implies that manual fire protection systems, like handheld extinguishers, are sufficient, while "Very High" scores necessitate the simultaneous application of multiple fire safety measures. This classification system allows hospitals to prioritize areas for intervention based on quantitative risk assessment.

**Table 2:** The recommended fire control actions on the basis of the calculated fire risk level.

Fire Risk Score	Fire Risk Level		Control Measures
≤1	**Acceptable**	Low	The use of manual fire protection systems and general methods, such as handheld fire extinguishers are recommended. It may sometimes be needed to apply extra actions to protect the occupant.
>1 to ≤1.6	**Unacceptable**	Moderate	The employment of the fire alarm system is suggested. It may sometimes be required to provide sufficient water supplies and the adoption of supplementary actions to preserve the occupants.
>1.6 to ≤4.5		High	The application of fire alarm and extinguishing systems, such as sprinklers, is necessary. If the risk level is ≥2.7, sufficient water supply should be ensured.
>4.5		Very High	Several approaches should be simultaneously implemented to decrease the fire risk level. The protection criteria as mentioned above are ineffective alone.

Risk interpretation*.

**1st risk level:** Acceptable risk: ≤1 (Green).

**2rd risk level:** Corrective measure is necessary: >1 to ≤1.6 (Yellow).

**3th risk level:** As soon as possible corrective measure should be considered: >1.6 to ≤4.5 (Orange).

**4th risk level:** Stopping the activity and corrective measure should be considered immediately: >4.5 (Red).

#### 2.3.5 Influential factors to the fire risk

We obtained the rate of contribution to the risk level as the difference of estimated fire risk values R assigned to the current and standard levels of the sub-parameters. According to the equations I-III, if the scores of potential risks (P), acceptable risk level (A), and protection level (D) were equal to 1, the fire risk score (R) will also be equal to 1. If the scores of P>1 or A, and D <1, then the fire risk score (R) is greater than the 1 and the safety is evaluated in a non-standard state. Accordingly, the q, v, and, z scores obtained from equation VI-IV as sub-parameters of p and p_1_ with a score higher than 1, were considered as effective factors. Besides, the identification of sub-parameters based on the scores A, A_1_, A_2_ obtained from equation I-III showed that the sum of the values of a, t, c, r, d should be less than 0.6. Therefore, sub-parameters with a score of ≥1 were identified as influencing factors (equation VII).

### 2.4. Ethical approval

The study protocol was reviewed and approved by the Research Ethics Committee of Semnan University of Medical Sciences (IR.SEMUMS.REC.1397.236). All interviews and data collection procedures adhered to the ethical guidelines described by this committee.

Prior to participation informed consent was obtained from all participants. Consent was provided in written form, ensuring that participants were fully informed about the study’s objectives, data collection processes, and confidentiality measures. Participants were made aware of their right to withdraw from the study at any stage without any consequences. For interviews, written consent was supplemented by verbal consent when appropriate particularly during onsite discussions. Verbal consent was documented in writing by the researcher with a witness confirming the participant’s agreement. This study involved only adult staff members (facility managers, maintenance staff, and ward supervisors) who voluntarily participated.

## 3. Results

Overall, of 84 assessed fire risks in three levels of building and their contents, occupants, and activities, the frequency and severity of safety hazards of 55 exceeded the acceptance levels in two hospitals. The majority (85.7%) of wards obtained non-acceptable risk evaluation at risk level of buildings, occupants or activities, and only 4 (14.28%) examined department had an acceptable risk in three mentioned levels. Overall, around 40.47% and 90.47% of health-care compartments scored a high-risk level in hospital A and B, respectively.

The Table 3 shows the risk levels vary across different wards. The highest risk levels are related to occupants for hospital A in general medicine (men’s), followed by cardiac surgery, whereas the pharmacy unit in hospital A had relatively low risk levels across all three exposure domains. In hospital B, the pediatrics and obstetrics and gynecology (OBGYN) units rated "very high" risk levels across all three domains, while the laboratory ward has relatively low risk levels. Surgery and emergency rooms obtained "very high" risk levels for the buildings and occupants. In hospital B, most spaces scored "very high" levels of risk for the exposure of occupants.

**Table 3:** Final levels of risks evaluated according to the exposure of R, R_1_, R_2_ in the various units of hospital A and B.

Hospital A	Exposure domains			Hospital B	Exposure domains		
Units	R	R_1_	R_2_	Units	R	R_1_	R_2_
Neuropsychiatry	0.822	1.221	0.331	Neonatal Intensive Care Unit (NICU)	0.576	2.982	0.245
Radiology	0.673	0.941	0.263	Surgical gynecology	2.605	4.547	1.135
Dialysis	0.556	0.986	0.185	Laboratory	0.889	1.934	0.635
Pharmacy	0.317	0.835	0.134	Pediatrics	6.730	9.452	10.76
General medicine (Men’s)	4.215	23.135	0.736	Obstetrics and Gynecology (OBGYN)	6.736	8.258	9.789
General medicine (Women’s)	1.474	2.342	0.602	Operating Room (OR)	4.938	9.005	2.074
Men’s surgery	1.263	1.348	0.521	Emergency	5.535	7.731	2.398
Cardiac surgery	1.455	14.452	0.598	Radiology	3.567	4.913	1.524
Gynecology surgery	1.302	1.343	0.545	Gynecological Intensive Care Unit	3.115	5.104	1.006
Meeting room	1.512	2.612	0.624	Speech therapy	3.112	5.921	1.004
Laboratory	0.574	0.905	0.189	Occupational therapy	3.116	5.110	1.007
Medical Intensive Care Unit (MICU)	0.671	2.134	0.273	Neonatal Unit (NNU)	3.114	5.103	1.005
Surgical Intensive Care Unit (SICU)	0.302	2.613	0.301	Administrative office	3.117	5.113	1.008
Emergency	2.283	3.331	0.725	Reception area	3.105	5.956	1.002

R: Buildings and contents, R_1_: Occupants, R_2_: Activities.

### 3.1. The buildings fire risk assessment

Our results showed the fire risk level of buildings and content (R) in 7 wards (50%) and 12 (85.71%) units were higher than the reference level (R>1) in hospitals A and B, respectively. The general medicine (Men’s) ward had the highest fire load in hospital A (4.215) mainly consisted of the location of the unit on the upper floors of the hospital building and the high density of patients with limited mobility. Based on the calculated building’s fire risk level of general medicine unit (R>1.9), the expected destruction would be about 80%-100% in the event of a fire. The structure of building in about 29% of health-care spaces compose of combustible materials. Furthermore, the risk level of fire in the emergency, meeting room, and surgical intensive care unit (SICU) exceeded the acceptable level. The expected damage levels of mentioned units in the case of fire also, will be 80%, 30%, and 10%-30%, respectively.

Obstetrics and gynecology (OBGYN) units scored the highest fire risk level (6.736) for building and its contents (R) in hospital B. The insufficient number of emergency exit mainly contributed to the high estimation of risk to buildings. Also, the pediatrics, emergency, operating room (OR), radiology, administrative office, occupational therapy, gynecological intensive care unit, neonatal unit (NNU), speech therapy, and reception area were in the next unacceptable ranks, respectively. The building’s fire risk levels of mentioned units (R> 1.9), indicate that 80%-100% of the existing compartments will be destroyed in the case of a fire incident in hospital B.

### 3.2. The occupants risk assessment

Our results showed the fire risk level of occupants (R_1_) for the majority of wards of the hospital A was greater than one that needs immediate corrective measures but in the departments of laboratory, pharmacy, dialysis, and radiology is less than 1 which was acceptable. The estimation of deterministic fire risk in terms of occupants in the Emergency ward, SICU, and medical intensive care unit (MICU) was 3.331, 2.612, and 2.134 respectively; which indicated hazards with a high-risk level. Additionally, our findings in hospital B revealed that fire risk scores for over 78% of the examined units were ≥4.5, and for 21.42% of wards ranged between 1.6 to 4.5. In addition, the pediatrics section scored the highest fire risk level in terms of occupants (9.452) compared to the other health-care occupancies. Increased demands for oxygen-supplying equipment and high density of ventilators as effective factors in increasing the risk of fire in the mentioned units. Moreover, Incapability of patients and decreased reaction to move in emergency conditions and escape from a fire area were identified as risky behaviors in a fire incident. More than one-third of occupants were not able to evacuate without help from the staff. Above two-third of occupants reported low degree of familiarity concerning the exit paths, safe locations and building arrangement.

### 3.3. The activities risk assessments

The fire risk level of activities in all studied wards of hospital A was acceptable. However, only 2 (14.28%) units of hospital B that including laboratory and Neonatal Incentive Care Units (NICU) obtained acceptable value of fire risk activities (R≤1). Thus, the required level of protection (D_2_) for activities is acceptable only in the laboratory and NICU wards. Overall, the fire risk scores of activities in hospital B showed that 57.14% wards were moderate level, and 14.28% of study units were high and very hard, respectively. Factors affecting the odds of fire in different units of hospital B mainly consisted of the high density of flammable materials, undeveloped educational programs regarding fire control and prevention, for worn electrical installations, and without protection.

### 3.4. The influential factors of risk assessment

Each unit’s influential fire risk factors (IFRF) include Fire Load Factor, Venting Factor, Access Factor, and Activation Factor. As shown in Table 4, the contribution rates (CR) of these factors vary across the wards, indicating the varying fire risk levels in different areas of Hospital A. The fire load factor had the highest contribution rate of influential fire risk factors, followed by the venting factor in the assessment of P and, P_1_ scores in all units of hospitals A and B.

**Table 4:** The contribution rate of influential fire risk factors in the studied wards of hospital A and B.

Exposure domains		R				R_1_				R_2_
Main indices		P			A	P_1_			A_1_	A_2_
		Sub-Parameters								
Type of hospital		Fire Load Factor (6.06)	Venting Factor (1.07)	Access Factor (0.08)	Activation Factor (0.1)	Fire Load Factor (6.06)	Venting Factor (1.07)	Access Factor (0.08)	Activation Factor (0.1)	Activation Factor (0.1)
	Units	CR	CR	CR	CR	CR	CR	CR	CR	CR
**Hospital A**	Neuropsychiatry	5.06	0.07	-0.91	-0.5	5.06	0.07	-0.91	-0.5	-0.5
	Radiology	5.06	0.07	-0.92	-0.5	5.06	0.07	-0.92	-0.5	-0.5
	Dialysis	5.06	0.07	-0.95	-0.5	5.06	0.07	-0.95	-0.5	-0.5
	Pharmacy	5.06	0.07	-0.96	-0.5	5.06	0.07	-0.96	-0.5	-0.5
	General medicine (Men’s)	5.06	0.07	-0.77	-0.5	5.06	0.07	-0.77	-0.5	-0.5
	General medicine (Women’s)	5.06	0.07	-0.77	-0.5	5.06	0.07	-0.77	-0.5	-0.5
	Men’s surgery	5.06	0.07	-0.88	-0.5	5.06	0.07	-0.88	-0.5	-0.5
	Cardiac surgery	5.06	0.07	-0.91	-0.5	5.06	0.07	-0.91	-0.5	-0.5
	Gynecology Surgery	5.06	0.07	-0.88	-0.5	5.06	0.07	-0.88	-0.5	-0.5
	Meeting room	5.06	0.07	-0.85	-0.5	5.06	0.07	-0.85	-0.5	-0.5
	Laboratory	5.06	0.07	-0.97	-0.5	5.06	0.07	-0.97	-0.5	-0.5
	Medicine Intensive Care Units (MICU)	5.06	0.07	-0.96	-0.5	5.06	0.07	-0.96	-0.5	-0.5
	Surgical Intensive Care Units (SICU)	5.06	0.07	-0.96	-0.5	5.06	0.07	-0.96	-0.5	-0.5
	Emergency	5.06	0.07	-0.82	-0.5	5.06	0.07	-0.82	-0.5	-0.5
**Hospital B**	National Intensive Care Units (NICU)	5.06	-0.66	-0.96	0	5.06	-0.66	-0.96	0	0
	Surgical gynecology	5.06	-0.06	-0.96	0	5.06	-0.06	-0.96	0	0
	Laboratory	5.06	-0.26	-0.97	0	5.06	-0.26	-0.97	0	0
	Pediatrics	5.06	-0.10	-0.92	0	5.06	-0.10	-0.92	0	0
	Obstetrics and Gynecology (OBGYN)	5.06	0.07	-0.92	0	5.06	0.07	-0.92	0	0
	Operation Room (OR)	5.06	0.07	-0.88	-0.5	5.06	0.07	-0.88	-0.5	-0.5
	Emergency	5.06	-0.03	-0.96	-0.5	5.06	-0.03	-0.96	-0.5	-0.5
	Radiology	5.06	0	-0.96	-0.5	5.06	-0.14	-0.96	-0.5	-0.5
	Gynecology Intensive Care Units	5.06	-0.05	-0.93	-0.5	5.06	-0.05	-0.93	-0.5	-0.5
	Speech Therapy	5.06	-0.05	-0.93	-0.5	5.06	-0.05	-0.93	-0.5	-0.5
	Occupational Therapy	5.06	-0.05	-0.93	-0.5	5.06	-0.05	-0.93	-0.5	-0.5
	Neonatal Units (NNU)	5.06	-0.05	-0.93	-0.5	5.06	-0.05	-0.93	-0.5	-0.5
	Administrative office	5.06	-0.05	-0.93	-0.5	5.06	-0.05	-0.93	-0.5	-0.5
	Reception area	5.06	-0.05	-0.93	-0.5	5.06	-0.05	-0.93	-0.5	-0.5

R: Buildings and contents, R_1_: Occupants, R_2_: Activities, P: Potential Risk, A: Acceptable Level, CR: Contribution Rate.

Based on the results the fire load factor and venting factor had the highest contribution rate following in the evaluation of P and, P_1_ scores in the all units of hospital A, respectively. Neuropsychiatry ward showed the highest contribution rate of fire risk factors, followed by other wards like radiology, dialysis, and pharmacy. Additionally, the investigation of the effective sub-parameters in hospital B showed fire load factor had the highest contribution to the fire risk with a numerical value of 5.06. However, the majority units of hospital B except OBGYN and OR units obtained acceptable scores of venting factors. According A, A_1_, and A_2_ values, the activation factors had not contribute to the occurrence of fire risk (A, A_1_, and A_2_<0.6).

### 3.5. Summary of results

A comprehensive assessment of fire risk levels was conducted across multiple wards in two hospitals. The evaluated risks are categorized into three domains: building and contents (R), occupants (R_1_), and activities (R_2_). The findings reveal that in hospital A, approximately 40.47% of compartments scored a high fire risk, while in Hospital B, this number reached 90.47%. Occupant-related risks were notably higher in hospital B, particularly in pediatric and OR units. In contrast, wards such as the pharmacy and laboratory in both hospitals displayed relatively low fire risk levels across all three domains. The Table 5 summarizes the final risk levels for each ward based on the three exposure domains in both hospitals.

**Table 5:** Summary of fire risk categories, key findings for hospitals A and B, and control measures based on the risk assessment analysis.

Category	Key Findings	Hospital A	Hospital B	Control measures
R	Severe fire risk due to building structures and combustible materials in both hospitals, especially in upper floors and high-patient-density wards.	Half of wards show unacceptable risk, with severe impact expected in case of fire (e.g., 80% destruction in high-risk areas).	85.7% of units show unacceptable risk, especially in OBGYN, pediatrics, and emergency units. Expected damage could be 80–100% in the case of fire.	- Strengthen fire resistance of building materials, especially in high-risk wards. - Increase emergency exits in high-risk areas (e.g., medical units). - Regular inspection and improvement of fire compartmentalization.
R_1_	Occupant evacuation is a critical issue in both hospitals, especially in wards with limited mobility patients (Pediatrics, ICU) and high oxygen use.	General medicine (Men’s) and cardiac surgery units exhibit very high risk (R1≥4.5). Around one-third of occupants may struggle to evacuate during emergencies.	Over 78% of wards have a very high risk (R1 ≥ 4.5), with the highest being Pediatrics (9.452). Occupant evacuation is highly problematic in these areas.	- Develop evacuation plans and provide fire drills, especially for high-risk units. - Increase staffing for patient assistance during evacuation. - Ensure fire alarms are easy to hear and accessible to all occupant
R_2_	Hospital B faces significant activity-related fire risks, primarily due to inadequate fire control systems and unprotected electrical installations.	Acceptable risk across all units (R2 ≤ 1).	Only 14.28% of wards show acceptable risk levels (e.g., Laboratory, NICU). High activity risk in most wards due to fire-prone activities.	- Improve maintenance of electrical systems. - Conduct training on fire control and prevention programs. - Limit storage of flammable materials in high-risk areas. - Implement regular fire prevention audits and updates to fire safety protocols.
IFRF	Fire load factor contributes the most to overall risk in both hospitals, highlighting the need for load reduction and improved ventilation systems in key wards.	Fire load and venting factors drive the highest risk levels across all units, with the most severe issues in high-density areas like general medicine.	Fire load and activation factors showed the non-acceptable and acceptable risk level, respectively across all units. Venting factors in most units except OBGYN and OR lead to reduced fire control capacity.	- Install and maintain effective fire suppression systems. - Focus on improving venting systems to reduce risk. - Enhance the fire load management by better controlling combustible materials.

R: Buildings and contents, R_1_: Occupants, R_2_: Activities, IFRF: Influential Fire Risk Factors.

## 4. Discussion

### 4.1. Overview of key findings

This study assessed the fire risk factors in healthcare facilities, focusing on two university hospitals. This study focused on the risk levels of buildings, occupants, and activities in various wards. The findings revealed that fire risks in healthcare facilities are significantly influenced by hospital layout, staff training, fire protection systems, and the presence of vulnerable patient populations. Our study demonstrated the effectiveness of using the Fire Risk Assessment Method for Engineering (FRAME) in identifying and mitigating fire risks across different wards in hospital environments. While previous studies have applied FRAME in specific healthcare settings [5,30,31,34], this study contributes new insights by broadening the scope of the assessment to include a wide range of hospital environments and identifying differences in fire risk between wards. The assessment identified that a majority of units in both hospitals exhibited risks at relatively high levels, particularly in terms of buildings and their contents, occupants, and activities. Specifically, pediatrics and OBGYN units were found to have higher risk level scores than other hospital sections. In terms of occupants, general medicine (men’s) and cardiac surgery units were identified as having high-risk levels, attributed to factors such as difficulty in patient mobility and increased use of oxygen-supplying equipment. Furthermore, the assessment highlighted those certain activities within the hospitals, such as laboratory and NICU, had acceptable risk levels, while others, such as operating and emergency rooms, exhibited high-risk levels for buildings and occupants.

### 4.2. Risk areas and contributing factors

Our analysis goes beyond previous studies by identifying several new risks in hospital environments. Specifically, we found significant risks associated with wards where patient immobility is prevalent (ICUs and emergency rooms), and where oxygen therapy is commonly used. These risks were amplified by the structural design issues, such as limited emergency escape routes and high-occupancy periods. This finding emphasizes the importance of integrating both architectural and operational controls into fire safety planning, which has not been thoroughly explored in prior risk assessments. The building layout, especially in older hospital wings, often lacks modern fire safety infrastructure, such as wide emergency exits and smoke barriers. These deficiencies not only complicate evacuation procedures but also elevate the risk of fire spreading through interconnected hospital units, particularly during high-occupancy periods [16,27].

Furthermore, our results highlight previously underreported high-risk wards, such as the pediatrics, OBGYN, and surgical gynecology units, suggesting that fire risks are not confined to areas traditionally thought to be hazardous [30]. For example, the high fire risk in OBGYN units due to inadequate fire protection systems and suboptimal staff training represents a critical gap in current fire safety practices, particularly in healthcare facilities with vulnerable patient populations. Improving staff readiness through regular fire drills and emergency preparedness training, especially in departments with vulnerable patient populations, can help in reducing fire risks. Lack of such preparedness has been identified as a factor in increasing fire hazards, as insufficient staff training and preparedness may delay effective response in emergency situations [35]. While existing studies [36,37] have documented fire risks in hospitals, particularly related to patient mobility and oxygen usage, our study contributes additional evidence of high fire risks in non-ICU wards. The identification of risks in previously overlooked departments highlights the necessity of hospital-wide fire safety strategies rather than focusing solely on critical care areas. Additionally, our results provide concrete examples of how older buildings, which lack modern fire detection and suppression systems, exacerbate fire risks. Previous studies have mentioned structural deficiencies in older facilities [27], but our study offers detailed risk quantifications that emphasize the need for updating infrastructure and improving compartmentalization to control fire spread.

This study found that the men’s general medicine (4.215) and OBGYN (6.73) had the highest risk levels for the buildings and contents in hospital spaces. Furthermore, hospital compartments including emergency, meeting room, and cardiac surgery units scored unacceptable risk levels. Our assessment indicated that the protection level of these units was found to be unacceptable with an expected level of destruction during a fire ranged from 20% to 100%. This study identified geometrical issues with building structures such as compartment higher than ground floor and limited availability of emergency escape routes. Previous case studies have highlighted the complexity of buildings such as the proximity of compartments with potential hazardous environments in Iranian healthcare settings [5,27].

### 4.3. Comparative analysis of findings

The comparison of our results with existing studies (Table 6), such as those by Danzi et al. (2021) and Nouri et al. (2022), confirms that fire risks are particularly acute in ICU and emergency wards due to low patient mobility and insufficient escape routes [36,37]. Our results align with Danzi et al., findings that immobile patients in ICU wards combined with poor escape planning cause serious fire hazards [36].

**Table 6:** Comparative analysis of fire risk factors in healthcare facilities for present study and existing literature: Methods, focus Areas, and key findings compared with current study.

Study	Method	Focus Area	Key Findings	Comparison with Current Study
**Wood et al. (2021)** [16]	General fire assessment	COVID-19 oxygen-related fires	Identified risk of fires due to high oxygen use in hospital settings treating COVID-19 patients.	Our study confirms this and highlights additional risks in wards like ICU and emergency rooms, particularly in Hospital B.
**Senin et al. (2022)** [26]	Fire safety assessment	Hospital fire safety awareness	Found a lack of fire protection systems and insufficient staff training in Malaysian public hospitals, leading to high fire risks.	Similar findings on lack of fire training and poor infrastructure in pediatric, ICU, and emergency wards in both studies.
**Kurd et al. (2021)** [34]	FRAME method	Fire pathology in hospitals	Fire risk levels in hospitals were high due to building design issues, flammable materials, and lack of adequate firefighting tools.	Our study also identified high fire risks in older buildings and poor emergency escape routes in Hospital A.
**Omidvari et al. (2020)** [38]	FMEA + decision-making	Fire risk in healthcare settings	Found that fire risks could be mitigated by improving material management and regular maintenance of equipment.	Our study suggests similar control measures, such as maintaining electrical equipment and proper material storage in hospitals.
**Danzi et al. (2021)** [36]	Qualitative assessment of ICU fire risk	ICU wards	High fire risk due to low patient mobility, inadequate escape routes	Similar findings for ICU and emergency units in the current study, where patient immobility is also a critical risk.
**Nouri et al. (2022)** [37]	FRAME method for fire risk evaluation	Fire protection systems and staff training in hospital wards	Increased fire risk due to inadequate fire protection systems and untrained staff	Pediatric and gynecology wards in the current study showed similar risks due to insufficient fire systems and training.
**Nikeghbal et al. (2021)** [39]	Occupant load analysis	Hospital population density and fire safety during extended hours	Increased population density heightened fire risks, particularly during extended working hours	The current study also observed elevated risks in high-occupancy areas like emergency and ICU during peak periods.
**Aslani et al. (2019)** [27]	FRAME method	Fire safety systems in older hospital wards	Found inadequacies in emergency systems in older hospital buildings	Similar results were found in the current study regarding older hospital wards with insufficient emergency preparedness.

Similarly, Nouri et al. (2022) highlighted inadequate fire protection systems and a lack of staff training as critical risk factors, which is consistent with our findings in pediatric and OBGYN units, where fire safety systems were insufficient, and training was suboptimal [37]. Proper training and equipment remain essential to mitigate these risks, as our results document the necessity of supporting fire preparedness in these units.

Occupancy levels and the design of healthcare facilities also play a vital role in fire safety. Nikeghbal et al. (2021) found that high population density in hospitals during extended working hours significantly heightened fire risks [39]. Our findings showed this observation, particularly during peak times in emergency and ICU wards, where the high volume of patients and staff complicates evacuation efforts in case of a fire. These densely populated areas require more stringent fire risk assessments and emergency protocols.

Furthermore, oxygen therapy equipment presents a significant fire hazard. Heraty Wood (2021) identified fire risks associated with oxygen-related equipment in Covid-19 hospitals [16]. In line with this, our study found that both ICU and emergency wards which heavily rely on oxygen therapy, are particularly susceptible to fires caused by oxygen enrichment. This highlights the need for fire prevention strategies in areas where oxygen therapy is routinely used.

Finally, older hospital wards face additional challenges in maintaining fire safety standards. Aslani et al. (2019) noted deficiencies in fire safety systems in older buildings, a problem also observed in our study [30]. Older hospital wards in both hospitals we examined were less equipped with modern fire detection and suppression systems, increasing their vulnerability to fire incidents. Enhancing fire safety systems in older healthcare facilities should be a priority to prevent fires and ensure patient and staff safety.

In extending our comparisons with studies such as those by Danzi et al. (2021) [36], Nouri et al. (2022) [37], and Aslani et al. (2019) [30], we emphasize that similar themes emerge in the literature, reinforcing our findings on fire risks in high-occupancy wards, oxygen-enriched environments, and older hospital buildings. This alignment with other research highlights the widespread nature of these fire risk factors across various healthcare settings and emphasizes the importance of comprehensive fire risk management strategies in hospitals.

Furthermore, comparing our results with additional studies (Danzi et al. 2021, Nouri et al. 2022, and Nikeghbal et al. 2021) supports the importance of improving fire protection systems, staff training, and patient mobility in high-risk wards, while also addressing high-occupancy periods and extended working hours that contribute to fire risks [36,37,39]. The need for building redesign and enhanced escape routes, similar to the suggestions by Heraty Wood (2021) and Aslani et al. (2019), further highlights the necessary steps to improve fire safety standards in healthcare facilities [16,30].

The current study’s findings contribute to the growing body of evidence regarding fire risk management in healthcare facilities, particularly in high-risk areas like ICUs, emergency rooms, and older buildings. Further research should explore additional preventative measures and updated regulations to mitigate these risks effectively.

### 4.4. Practical implications and recommendations

Based upon fire risk factors identified in our analysis we recommend that hospitals establish regular fire safety training sessions appropriate for the specific departments, enhancing staff readiness to respond to potential fire incidents. Furthermore, upgrading safety infrastructure, including fire detection and suppression systems, in high-risk areas such as emergency and intensive care units could mitigate risks associated with the building layout and high equipment density. Ensuring clear, unobstructed evacuation routes and routine checks on emergency exits are also crucial in adapting hospital design for safe and rapid evacuation.

### 4.5. Future risk considerations

The changing nature of healthcare settings, especially during crises such as pandemics, can significantly alter fire risk factors. Increased reliance on temporary facilities, rapid installations of high-powered ventilation systems, and heightened use of oxygen equipment during respiratory-focused disease outbreaks (e.g., COVID-19) amplify fire hazards due to increased oxygen density and added electrical load. Recognizing these potential future risks is essential for adapting fire risk assessments and preparedness. Our study recommends integrating risk assessment practices that account for rapid changes in equipment use, facility layout, and staff capacity under unique conditions like pandemics.

## 5. Study limitations

A limitation of our developed risk basement method is the focus on specific hospitals and wards, which may restrict the generalizability of our findings to other healthcare facilities. The decision to focus on these two hospitals was guided by several practical considerations, including available resources, access permissions, and the time required for detailed fire risk assessments across multiple wards and compartments. Despite this limitation, these hospitals were selected as representative of medium- to large-sized facilities in similar regional settings. We propose that future research should focus on conducting comparative studies using the FRAME method in various workplaces and healthcare facilities to allow for comparisons and the identification of challenges and best practices in fire risk assessment and management. This approach can provide valuable insights into effective strategies for mitigating fire risks in different settings.

## 6. Conclusion

Based on the risk scores obtained it is evident that fire risk levels are unacceptable for majority of activities, occupants, and buildings within hospital units. High oxygen therapy requirements particularly for treating respiratory infections increased the risk of fire and explosion incidents in hospitals. Comparing the two hospitals Hospital A had a higher overall risk level due to its older building infrastructure and higher patient acuity levels. The newer Hospital B faced challenges in maintaining fire safety standards in wards with vulnerable occupants. This means preventive measures should be based on the unique characteristics of each hospital. specific risk factors such as fire load, venting, access, and activation factors are essential for developing fire safety measures to enhance preparedness and response capabilities.

This research has demonstrated that the developed fire risk assessment method can provide proactive approach, beyond identifying risk factors and levels, offers valuable control solutions for managing fire risks within hospital settings. Our data results not only aid in identifying key fire risk factors and their impact on safety measures but also help in planning effective interventions to prevent fires and enhance safety within the healthcare system. Fire safety management should improve staff training on fire safety protocols, conducting regular fire drills, and updating infrastructure to meet fire safety standards. future research should focus on validating the fire risk assessment method across different workplaces and conducting studies to track changes in fire risk factors to manage fire risk.

In summary, the FRAME method’s systematic approach, combined with its adaptability to specific hospital settings, offers a practical and efficient way to address fire and explosion hazards in hospitals. This study highlights the value of FRAME in both identifying existing fire risks and guiding the development of safer hospital environments.
